# RADAR-Septic Shock: A Population-Based Surveillance and Risk-Adjusted Benchmarking System for Septic Shock in Catalonia

**DOI:** 10.3390/medsci14060616

**Published:** 2026-09-29

**Authors:** Clara Reina-Aguilar, Juan Carlos Yébenes, Pedro Castro, Emili Vela, Josep M. Badía, Juan Carlos Ruíz-Rodríguez, Francesc Xavier Jiménez-Fábrega, Antoni Artigas, Carol Lorencio

**Affiliations:** 1 Intensive Care Department, Hospital Universitari de Mataró, 08304 Mataró, Spain; creinaag@csdm.cat; 2 Grup De Recerca en Sèpsia i Xoc Sèptic (GRSiXS), 08304 Mataró, Spain; jmbadia@fphag.org (J.M.B.); clorencio.girona.ics@gencat.cat (C.L.); 3 Doctoral Programme in Medicine of the Universitat de Girona, 17004 Girona, Spain; 4 Quality and Planning Department, Hospital Universitari de Mataró, 08304 Mataró, Spain; 5 Advisory Committee “Codi Sèpsia”, CatSalut, 08028 Barcelona, Spain; evela@catsalut.cat (E.V.); juancarlos.ruiz@vallhebron.cat (J.C.R.-R.); francescxavierjimenez@gencat.cat (F.X.J.-F.); aartigas@tauli.cat (A.A.); 6 Medical Intensive Care Unit, Hospital Clínic de Barcelona, 08036 Barcelona, Spain; pcastro@clinic.cat; 7 Division of Information Systems, Catalan Health Service, Department of Health of Catalonia, 08028 Barcelona, Spain; 8 Digitalization for the Sustainability of the Healthcare System (DS3), 08908 L’Hospitalet de Llobregat, Spain; 9 Department of Surgery, Hospital General Granollers, Universitat Internacional de Catalunya, 08402 Granollers, Spain; 10 Intensive Care Department, Vall d’Hebron University Hospital, 08305 Barcelona, Spain; 11 Shock, Organ Dysfunction and Resuscitacion Research Group, Vall d’Hebron Institut of Research (VHIR), 08035 Barcelona, Spain; 12 Operative Direction PLANUC (Pla Nacional d’Urgències de Catalunya), 08306 Barcelona, Spain; 13 Research Direction, Emergency Medical System (SEM), CatSalut, 08908 L’Hospitalet de Llobregat, Spain; 14 Institud d’Investigació i Innovació Parc Tauli (I3PT-CERCA), CIBERes, Corporacion Sanitaria Universitaria Parc Tauli, 08208 Sabadell, Spain; 15 Intensive Care Department, Hospital Universitari de Girona, Dr. Josep Trueta, 17007 Girona, Spain

**Keywords:** septic shock, population-based surveillance, risk-adjusted benchmarking, healthcare quality measurement, epidemiology

## Abstract

**Background:** We established RADAR-Septic Shock (Registry of Administrative Data Automatically Recorded for Septic Shock) as a population-based surveillance system to monitor the epidemiology, healthcare utilization, clinical outcomes, and risk-adjusted performance of septic shock across the Catalan publicly funded healthcare system. **Methods:** A retrospective population-based study was conducted using the CMBD-HA administrative database, including all public hospital discharges coded for septic shock (ICD-10-CM codes R65.21 and T81.12XA) in Catalonia between 2018 and 2024. Variables included demographic characteristics, comorbidities, source of infection, setting of acquisition, organ dysfunctions, and advanced organ support. Mortality risk was assessed using multivariable logistic regression, and model performance was evaluated using the area under the receiver operating characteristic curve (AUROC) and the area under the precision-recall curve (AUPRC). Hospital benchmarking was based on observed-to-expected (O/E) mortality ratios. **Results:** Between 2018 and 2024, RADAR-Septic Shock captured 37,855 episodes of septic shock. In 2024, 6522 episodes were recorded, corresponding to an incidence of 80.28 cases per 100,000 inhabitants per year. Overall, 79% of episodes were community-acquired and 21% hospital-acquired. Patients were predominantly male (60%), with a mean age of 68.7 years, and 57% had a high-risk comorbidity profile. Overall in-hospital mortality was 36.7%, increasing to 49.5% in hospital-acquired septic shock. Among patients with available APACHE II scores, mortality progressively declined across all severity categories over the study period, except during the COVID-19 pandemic. The mortality prediction model demonstrated acceptable discrimination (AUROC: 0.739; 95% CI: 0.726–0.752; AUPRC: 0.554). Risk-adjusted benchmarking identified substantial variation in outcomes across hospitals and territorial clusters. **Conclusions:** RADAR-Septic Shock provides a standardized population-based surveillance framework for monitoring the epidemiology, healthcare utilization, clinical outcomes, and risk-adjusted performance of septic shock. In Catalonia, it identified an annual incidence of approximately 80 cases per 100,000 inhabitants and documented improving mortality over time, except during the COVID-19 pandemic. By enabling risk-adjusted benchmarking across healthcare institutions, RADAR-Septic Shock may support quality-improvement initiatives, healthcare planning, and sepsis policy development.

## 1. Introduction

Sepsis is a life-threatening condition and a major public health challenge worldwide [1]. Improving surveillance, healthcare preparedness, and quality of care has therefore become a global priority, as reflected in the Global Agenda for Sepsis 2030 [2]. Septic shock, one of the most severe manifestations of sepsis, entails substantial morbidity, mortality, and healthcare resource utilization [1]. Continuous surveillance of its incidence, outcomes, and healthcare delivery is essential to support health system planning, identify opportunities for quality improvement, and benchmark institutional performance [3].

Recognizing this need, the recently updated Surviving Sepsis Campaign guidelines suggest that hospitals and healthcare systems implement structured “Code Sepsis” or “Sepsis Huddle” protocols as part of quality improvement strategies for sepsis care [4]. Several years before this recommendation, the Catalan Health Service (Servei Català de la Salut, CatSalut) designed a region-wide Sepsis Code to standardize early recognition, initial management, and interhospital coordination for patients with sepsis across the publicly funded healthcare system [5,6].

Training, audit, and systematic feedback of process and outcome indicators constitute key components of this regional strategy. CatSalut coordinates a tax-funded universal healthcare system serving approximately 8 million inhabitants in Catalonia, an autonomous region in northeastern Spain. This integrated healthcare organization provides a unique opportunity to implement population-based surveillance and risk-adjusted benchmarking across an entire regional healthcare network [7]. To support the continuous evaluation of the Sepsis Code strategy, CatSalut developed the Registry of Administrative Data Automatically Recorded for Septic Shock (RADAR-Septic Shock), a population-based surveillance system based on routinely collected administrative data from the Minimum Basic Data Set for Acute Care Hospitals (CMBD-HA), a mandatory administrative database covering all publicly funded acute care hospitals in Catalonia [8].

We hypothesize that an automated population-based surveillance system leveraging routinely collected administrative healthcare data can provide a robust and comprehensive characterization of the epidemiology, healthcare burden, and clinical outcomes of sepsis in Catalonia, while enabling the assessment of outcome variability across hospitals with different levels of complexity. In this first report of RADAR-Septic Shock, we describe the implementation of this regional surveillance system and its application to estimate the population incidence of septic shock, characterize patient profiles and healthcare utilization, and generate risk-adjusted benchmark indicators to support quality improvement across the Catalan healthcare system.

## 2. Materials and Methods

### 2.1. Study Design and Setting

This study reports the implementation and first results of the Registry of Administrative Data Automatically Recorded for Septic Shock (RADAR-Septic Shock), a population-based surveillance system developed by the Catalan Health Service (CatSalut) to monitor the epidemiology, outcomes, and quality of care of patients with septic shock across the regional healthcare system.

A retrospective population-based analysis of RADAR-Septic Shock data was conducted covering the period 2018–2024.

### 2.2. Regional Organization of Sepsis Care

Catalonia is an autonomous region in northeastern Spain with approximately 8 million inhabitants and a tax-funded universal healthcare system coordinated by CatSalut. Since 2015, sepsis care has been organized through a regional Sepsis Code network [6]. Hospitals are classified into three categories according to their capability to manage patients with sepsis: community hospitals without intensive care units (ICUs), general hospitals with ICUs, and high-complexity hospitals providing highly specialized care, including complex source control. Each hospital belongs to one of seven territorial referral networks (hereafter referred to as clusters), ensuring access to the appropriate level of care across the region [5,6].

RADAR-Septic Shock is based on the Minimum Basic Data Set for Acute Care Hospitals (CMBD-HA), a mandatory administrative database that collects standardized ICD-10-CM/PCS discharge information from all publicly funded acute-care hospitals in Catalonia, enabling population-wide surveillance and interhospital comparisons [8].

### 2.3. Study Population

Eligible cases were defined as hospital discharge records containing a primary or secondary diagnosis of septic shock (ICD-10-CM code R65.21) or postprocedural septic shock (T81.12XA) in patients with registered residency in Catalonia.

Temporal trends were evaluated over the period 2018–2024 using data from all eligible hospitals. Interhospital comparisons were restricted to institutions reporting more than 20 septic shock cases annually to ensure stable estimates. Data from the most recent complete year (2024) were used for risk-adjusted benchmarking analyses.

#### 2.3.1. Study Variables

The following variables were extracted from RADAR-Septic Shock: demographic characteristics (age and sex); selected clinically relevant comorbidities (cirrhosis, malignancy, and dementia); and episode-related variables, including source of infection, setting of acquisition (community- versus hospital-acquired), organ dysfunctions, and advanced organ support (mechanical ventilation, renal replacement therapy (RRT) and extracorporeal membrane oxygenation (ECMO). When available, APACHE II scores were recorded, and patients were stratified into three severity categories: moderate risk (APACHE II < 20), high risk (20–30), and very high risk (>30).

Microbiological information was derived exclusively from ICD-10 discharge codes recorded in the CMBD database. Due to the administrative nature of the dataset and the population-based benchmarking purpose of the model, specific pathogen identification codes were not incorporated. Multidrug-resistant microorganisms (MDRMs) were included as a binary variable based on the presence of an ICD-10 code indicating antimicrobial resistance during hospitalization.

To characterize the overall comorbidity burden, patients were also classified according to the Adjusted Morbidity Groups (AMGs) using data from the Catalan Health Surveillance System (CHSS). This population-based system integrates data from hospital admissions, primary care visits, emergency department encounters, long-term and specialized nursing facilities, palliative care services, and mental health services. It also incorporates prescription and pharmacy expenditure data, as well as billing records encompassing outpatient specialist visits, hospital-at-home activity, medical transportation (urgent and non-urgent), outpatient rehabilitation, respiratory therapies, and dialysis treatments [9].

#### 2.3.2. Outcomes

The primary outcome was in-hospital mortality. Secondary outcomes included hospital length of stay and risk-adjusted hospital performance, expressed as observed-to-expected (O/E) mortality ratios.

#### 2.3.3. Statistical Analysis

Descriptive analyses included absolute frequencies, percentages, and incidence rates per 100,000 inhabitants per year. Associations with in-hospital mortality were evaluated using multivariable logistic regression, whereas hospital length of stay was analysed using log-transformed linear regression because of its non-normal distribution. The discrimination of the mortality prediction model was assessed using the area under the receiver operating characteristic curve (AUROC).

Hospital performance was evaluated using the O/E mortality ratio. Expected mortality was estimated from the individual predicted probabilities generated by the multivariable logistic regression model. Confidence intervals (CIs) were calculated using the normal approximation for mortality indicators and the Breslow–Day method for efficiency indicators.

#### 2.3.4. Ethics

The study was approved by the Hospital Universitari de Mataró Clinical Research Ethics Committee (CEIm code CEIM_20/2018) on 21 March 2018, which granted a waiver for informed consent. The waiver was considered appropriate because this was a retrospective, population-based observational study using routinely collected administrative healthcare data. The study involved no direct participant contact, no intervention, and no modification of standard clinical care. Furthermore, all data were anonymized prior to analysis, and individual participants could not be identified. Given the large population covered and the retrospective nature of the study, obtaining informed consent from all individuals was considered impracticable and would not have been feasible without compromising the validity and completeness of the population-based analysis. The Ethics Committee determined that the research posed minimal risk to participants and met the criteria for exemption from informed consent. All study procedures were conducted in accordance with the Declaration of Helsinki and its subsequent amendments and with applicable data protection regulations. The project was registered on 9 September 2025, on ClinicalTrials.gov (NCT07174765).

This study is part of the PhD thesis of Clara Reina within the Doctoral Programme in Molecular Biology, Biomedicine and Health of the Universitat de Girona (UdG).

## 3. Results

### 3.1. Population Characteristics

**Figure 1:**
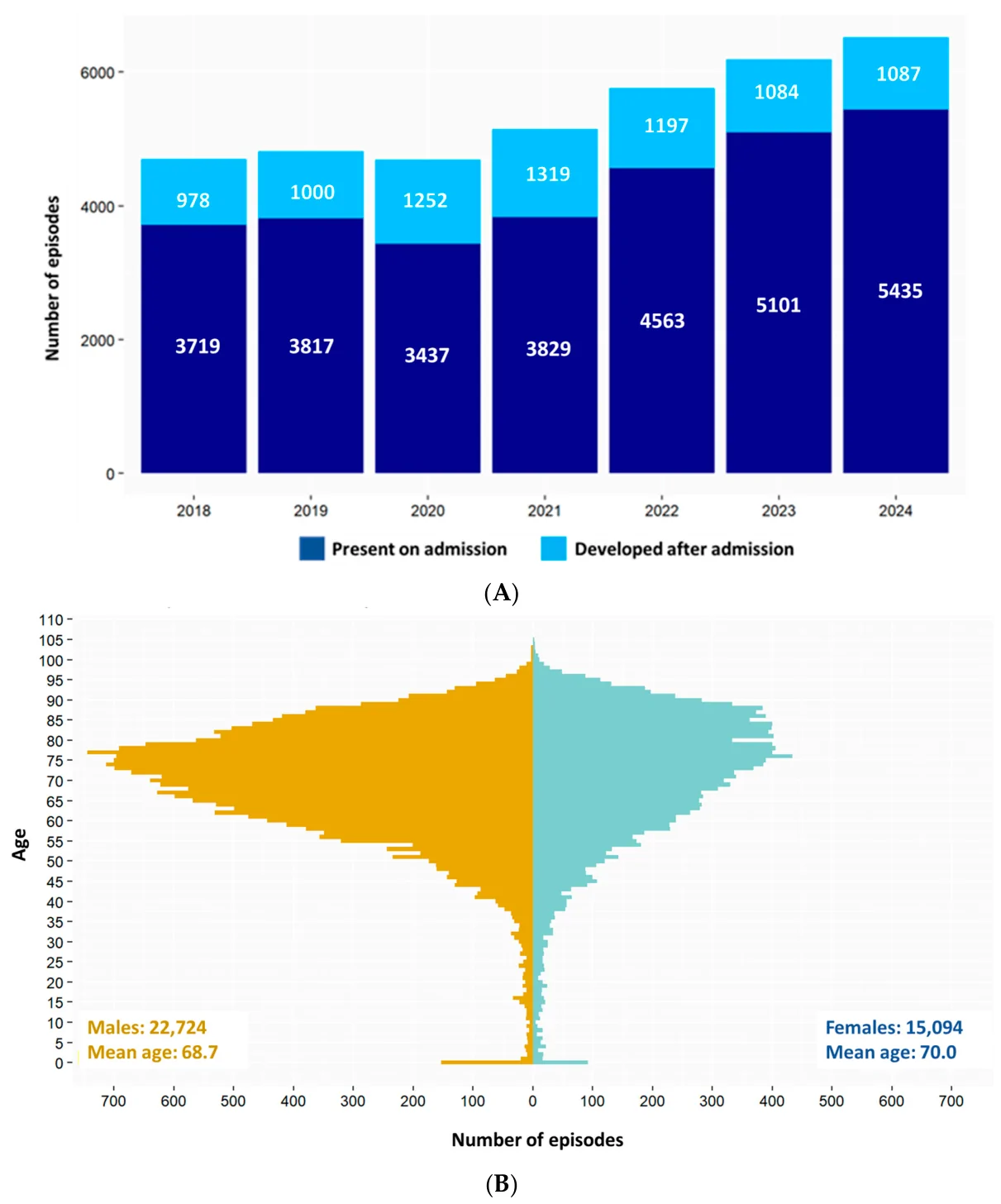

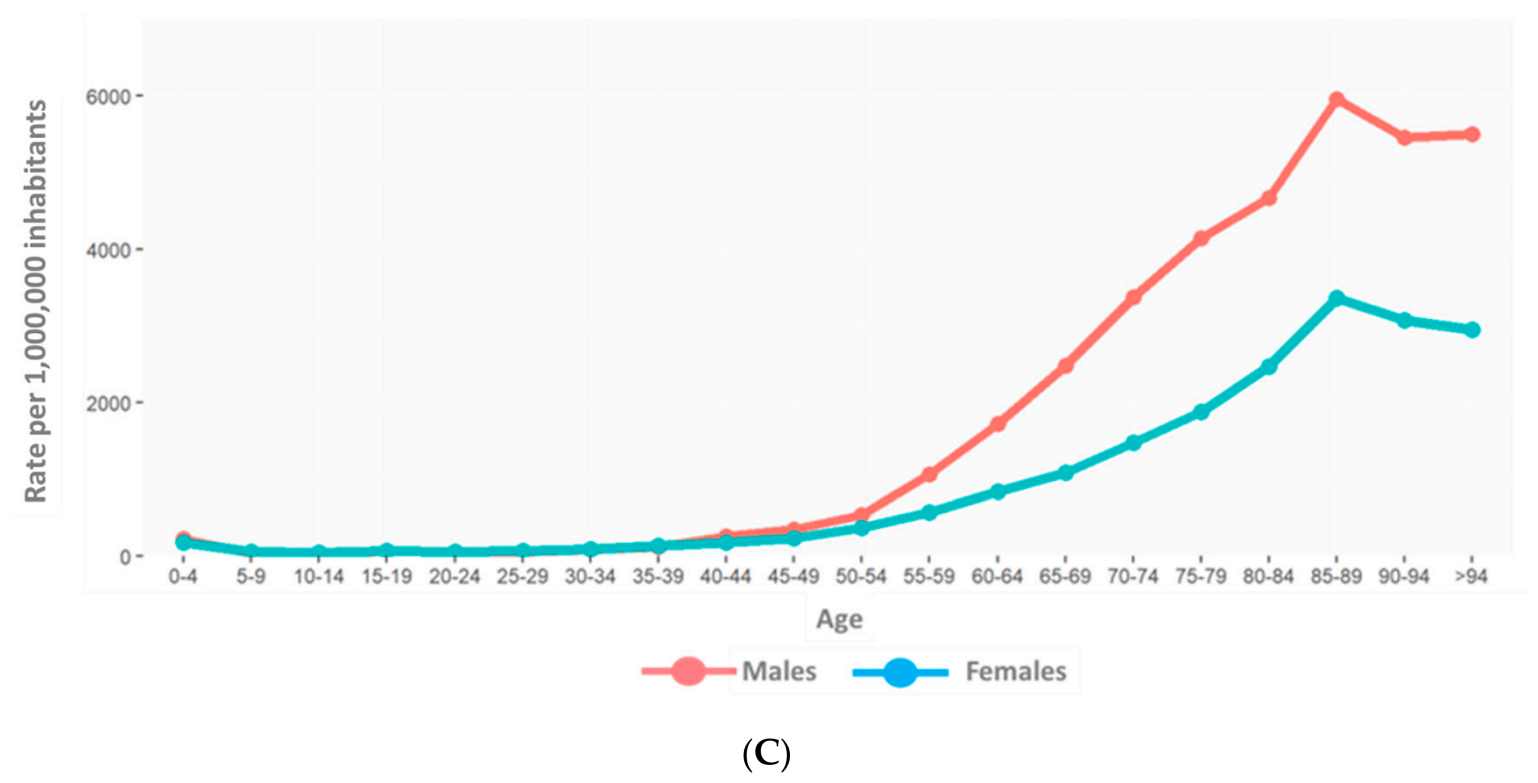
Characteristics of the population captured by RADAR-Septic Shock between 2018 and 2024. (**A**) Annual number of septic shock episodes according to setting of acquisition. (**B**) Age and sex distribution of the study population (**C**) Age- and sex-specific incidence of septic shock per million inhabitants.

Between 2018 and 2024, RADAR-Septic Shock captured 37,855 episodes of septic shock from 41 hospitals across Catalonia. The demographic characteristics of the study population are summarized in Figure 1. According to the Adjusted Morbidity Groups (AMGs), 57% of patients had a high-risk comorbidity profile, whereas only 16% belonged to the low- or baseline-risk categories. The most frequent comorbidities were diabetes (36%), malignancy (32%), chronic kidney disease (29%), heart failure (27%), and chronic obstructive pulmonary disease (COPD) (26%).

Episode characteristics are summarized in Table 1. Approximately 80% of septic shock episodes were community-acquired, although the proportion of hospital-acquired septic shock increased during the COVID-19 pandemic (2020–2021). The most common sources of infection were urinary, pulmonary, and abdominal. Multidrug-resistant (MDR) microorganisms were reported in 6.07% of cases. Source of infection, organ dysfunctions and the need for advanced organ support are also detailed in Table 1.

### 3.2. Clinical Outcomes

Overall in-hospital mortality was 36.7%. Mortality was substantially higher among patients with hospital-acquired than community-acquired septic shock (49.49% vs. 33.43%).

Mortality declined progressively throughout the study period, except during the COVID-19 pandemic.

**Figure 2:**
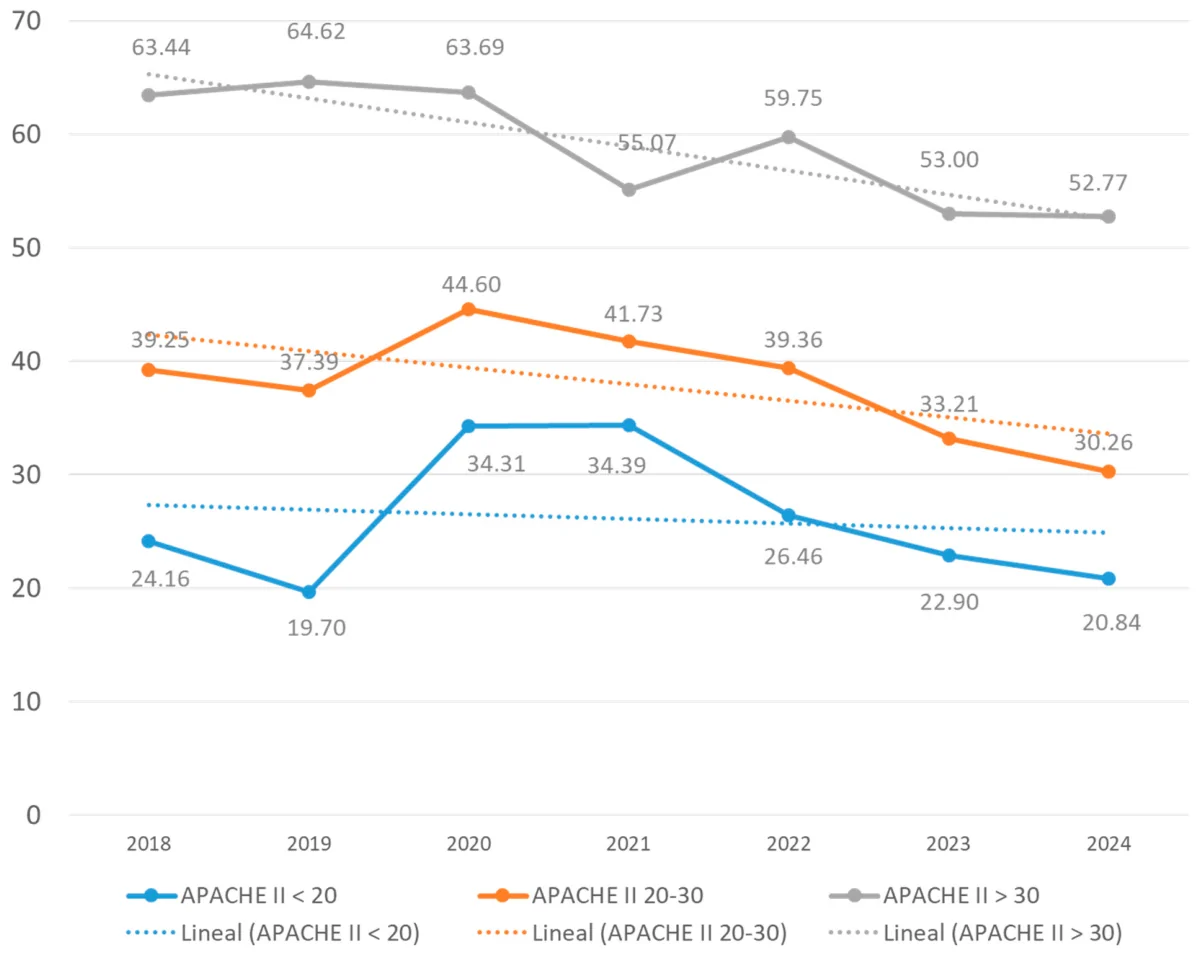
Temporal trends in in-hospital mortality stratified by APACHE II category: moderate risk (APACHE II < 20) (*p* < 0.001); high risk (APACHE II 20–30) (*p* < 0.001); very high risk (APACHE II > 30) (*p* = 0.022).

APACHE II scores were available for 10,029 episodes. Among patients with very high risk (APACHE II > 30), mortality progressively decreased between 2018 and 2024, with no apparent impact of the pandemic. In contrast, mortality temporarily increased during the pandemic among patients in the moderate- and high-risk APACHE II categories before declining to the lowest levels observed during the study period (Figure 2).

### 3.3. Benchmark Cohort (2024)

Hospital benchmarking analyses were performed using data from 2024, the most recent complete year available. During that year, RADAR-Septic Shock identified 6522 episodes, of which 83.3% were community-acquired (*n* = 5435) and 16.7% were hospital-acquired (n = 1087). Based on the reference population of 8,124,126 inhabitants (Institut Català d’Estadística [IDESCAT], 1 January 2025) [10], the overall incidence was 80.28 cases per 100,000 inhabitants per year, including 66.90 community-acquired cases and 13.38 hospital-acquired cases per 100,000 inhabitants per year.

Community hospitals without an ICU initially managed 14.37% of cases, general hospitals with an ICU managed 32.18%, and high-complexity hospitals managed 53.55%, reflecting the distribution of septic shock care across the regional sepsis network.

### 3.4. Risk-Adjusted Benchmarking

To enable risk-adjusted benchmarking across hospitals, a multivariable mortality prediction model was developed. The model demonstrated acceptable discrimination, with an AUROC of 0.739 (95% CI 0.726–0.752) and an area under the precision-recall curve (AUPRC) of 0.554 (Table 2).

**Figure 3:**
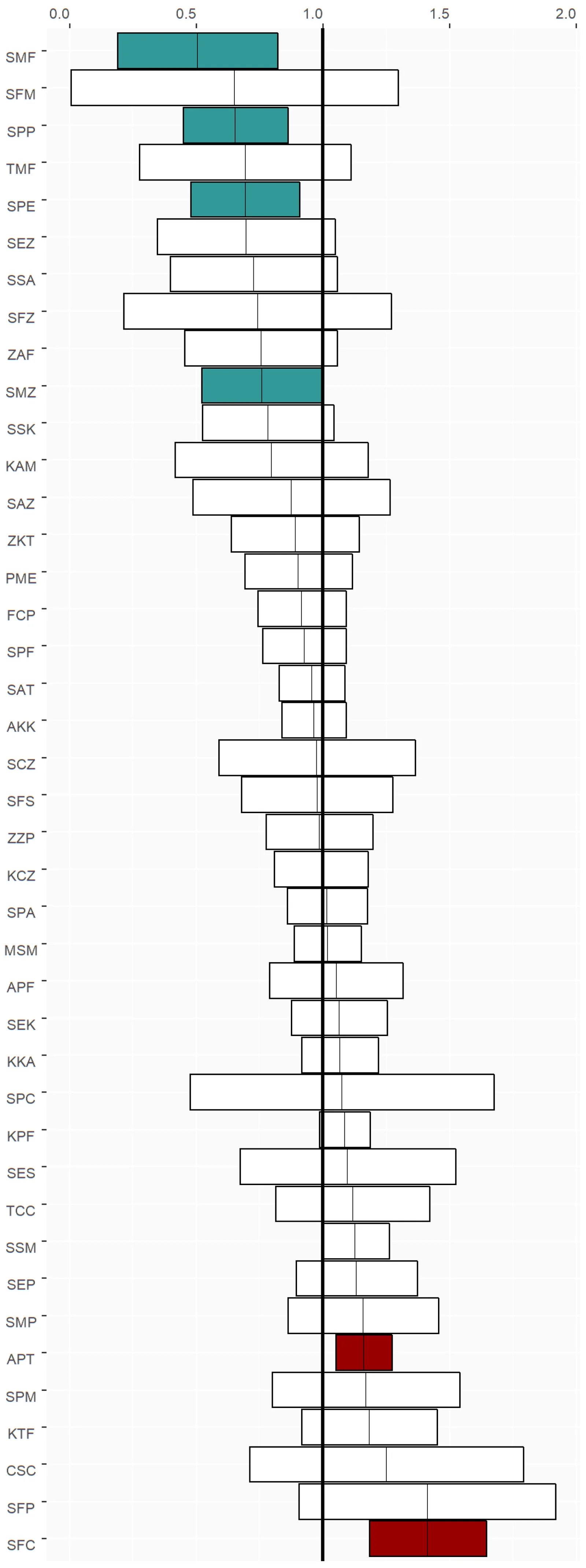
Risk-adjusted hospital performance according to the observed-to-expected (O/E) mortality ratio (RADAR-Septic shock benchmark cohort 2024). Hospitals are represented by anonymized alphabetical codes and ordered according to increasing O/E ratio. Boxes represent O/E estimates and 95% confidence intervals. Values below 1 indicate lower-than-expected mortality, whereas values above 1 indicate higher-than-expected mortality. Green boxes indicate hospitals with significantly lower-than-expected mortality, whereas red boxes indicate significantly higher-than-expected mortality.

Application of this model identified substantial variability in hospital performance across Catalonia, allowing hospitals to be stratified into performance quartiles according to their O/E mortality ratio (Figure 3). Two hospitals showed significantly lower-than-expected mortality, whereas two showed significantly higher-than-expected mortality. When results were aggregated according to the seven territorial referral networks, differences in risk-adjusted mortality were also observed between regional clusters (Supplementary Figure S1).

Hospital-level variability in O/E mortality was observed across all hospital complexity categories, including community, general, and high-complexity hospitals (Supplementary Figure S2).

## 4. Discussion

The present study describes the implementation of RADAR-Septic Shock, a population-based surveillance system designed to continuously monitor the epidemiology, clinical outcomes, healthcare utilization, and risk-adjusted performance of septic shock across the Catalan publicly funded healthcare system. Beyond providing contemporary epidemiological data, RADAR-Septic Shock enables continuous monitoring of clinical outcomes and healthcare utilization while supporting risk-adjusted benchmarking across hospitals and territorial Sepsis Code networks (clusters) using standardized administrative data, thereby supporting quality improvement initiatives and healthcare planning. The present analysis highlights four main findings: the high population burden of septic shock, its close association with ageing and comorbidity, the temporal changes in outcomes observed during the COVID-19 pandemic and over the study period, and the substantial variability in risk-adjusted mortality across hospitals.

Reliable estimates of the population incidence of septic shock remain scarce in Europe. Previous studies based on administrative databases have reported highly variable incidence rates, ranging from 39 to 171 cases per 100,000 inhabitants [11,12], probably reflecting differences in demographic characteristics, healthcare organization, and coding practices. Using a standardized regional registry, we estimated an annual incidence of approximately 80 cases per 100,000 inhabitants in Catalonia. Because private hospitals were not included, this figure may represent a slight underestimate of the true regional incidence.

Our results also demonstrate a strong relationship between septic shock and both age and comorbidity. The incidence doubled among individuals older than 65 years and exceeded 300 cases per 100,000 inhabitants in those aged over 75 years. Given the progressive ageing of industrialized societies, these findings have important implications not only for acute hospital care but also for post-ICU recovery, long-term functional outcomes, and healthcare resource allocation [9].

Approximately 80% of septic shock episodes were community-acquired, whereas one in five occurred after hospital admission. This distribution has important organizational implications. Moreover, a substantial proportion of patients are initially identified and managed in regional or community hospitals, emphasizing that early recognition, initial resuscitation, and timely activation of referral pathways should be implemented across the entire healthcare network rather than being restricted to tertiary or high-complexity hospitals. These findings further support the regional Sepsis Code strategy adopted in Catalonia [5,6].

The temporal analyses also yielded several noteworthy observations. First, the COVID-19 pandemic (2020–2021) was associated with a marked increase in the proportion of hospital-acquired septic shock together with a substantial deterioration in clinical outcomes, particularly among patients who developed septic shock during hospitalization. Similar collateral effects of healthcare system saturation have been reported for sepsis [13] and for other time-dependent conditions such as ST-elevation myocardial infarction and stroke [14,15,16]. Second, despite these temporary disruptions, in-hospital mortality progressively declined throughout the study period. The availability of APACHE II scores in a large subgroup of patients allowed for partial adjustment for disease severity and demonstrated that this improvement persisted across comparable severity categories. Particularly striking was the increase in mortality among patients with APACHE II scores below 30 during the pandemic, suggesting that structural pressures on healthcare delivery may disproportionately affect patients with potentially reversible disease.

The proportion of multidrug-resistant (MDR) pathogens remained stable throughout the study period at approximately 6%, which is lower than the figures reported in several previous cohorts (10–28.7%) [17,18,19]. Considering that approximately one-fifth of septic shock episodes were hospital-acquired, this finding may reflect incomplete microbiological coding or underreporting of MDR microorganisms within administrative databases. Because antimicrobial resistance has major clinical and therapeutic implications, improving the accuracy of MDR coding should constitute a priority for future surveillance initiatives.

Unlike many administrative studies of sepsis, which identify cases using combinations of infection and organ dysfunction codes, RADAR-Septic Shock relies on a single, specific ICD code for septic shock. This approach provides greater diagnostic specificity and makes septic shock particularly suitable for population-based surveillance using administrative data [20,21]. This observation is consistent with the study by Kadri et al. [22], who demonstrated that administrative coding for septic shock showed high specificity despite lower sensitivity when compared with clinically defined septic shock. Although differences in coding practices between institutions should always be considered, the CMBD-HA constitutes the standardized administrative database used throughout the Catalan publicly funded healthcare system for healthcare planning, activity monitoring, and resource allocation, providing a robust and homogeneous framework for population surveillance and comparative analyses [8].

One of the principal strengths of RADAR-Septic Shock is its integration within the regional Sepsis Code strategy. Continuous audit and systematic feedback are essential components of any quality improvement programme [7,23], particularly when implementation involves numerous hospitals with heterogeneous resources and organizational characteristics. By capturing more than 6000 episodes of septic shock annually, RADAR provides continuous information on incidence, patient characteristics, healthcare utilization, and clinical outcomes while simultaneously enabling risk-adjusted benchmarking across hospitals and territorial clusters. The hospital benchmarking analysis (Figure 3) exemplifies the practical value of this approach by providing an objective framework for identifying unwarranted variations in outcomes and prioritizing quality improvement initiatives. Furthermore, supplementary analyses (Supplementary Figures S1 and S2) demonstrate that this approach can also be extended to territorial clusters and hospitals with different levels of complexity This benchmarking capability is supported by the standardized structure of the CMBD-HA database and by the acceptable discriminative performance of the multivariable mortality model (AUROC: 0.739; 95% CI: 0.726–0.752; AUPRC: 0.554), which provides an appropriate framework for institutional risk adjustment despite the inherent limitations of administrative data.

Importantly, the associations observed in this model should not be interpreted as causal effects, as the primary aim of the study was to develop a risk-adjustment model for benchmarking septic shock outcomes rather than to establish causality. The association between multidrug-resistant microorganism (MDRM) isolation and lower mortality likely reflects survivor-time bias and residual confounding. MDRM detection may occur later during hospitalization and does not necessarily indicate that the resistant microorganism was responsible for the septic shock episode. Furthermore, the relatively low prevalence of MDRM coding in our cohort may have influenced the stability of this estimate.

Likewise, the lower mortality observed in genitourinary, device-related, and abdominal infections is consistent with the well-recognized prognostic heterogeneity of septic shock according to the site of infection. Compared with respiratory infections, these sources are often associated with earlier diagnosis and more effective source control, resulting in a more favourable prognosis [24,25,26]. In particular, the lower mortality observed in abdominal infections may be partly explained by the inclusion of biliary tract infections, which have been associated with better outcomes than other intra-abdominal sources because timely and effective source control is frequently achievable. Therefore, these findings should be interpreted as reflecting differences in case mix and underlying prognosis rather than a true protective effect.

Importantly, RADAR-Sepsis should not be interpreted as a clinical decision-support or diagnostic tool. Its primary purpose is to support healthcare planning, quality assessment, and optimization of clinical pathways. Hospitals with higher-than-expected mortality should therefore be encouraged to review adherence to evidence-based practices and monitor key performance indicators (KPIs) of sepsis care. In this context, the ongoing development of the complementary Catalan Sepsis and Septic Shock Registry (RSIXS) [19], designed to systematically capture KPIs derived from the Surviving Sepsis Campaign guidelines [4], represents a logical next step that will complement the benchmarking capability of RADAR with detailed process-of-care evaluation.

This study has several limitations. First, although the CMBD-HA is the standardized administrative database used across the Catalan publicly funded healthcare system, the correlation between administrative coding and clinically validated septic shock has not been specifically assessed within our setting. Consequently, the results should be interpreted with appropriate caution, particularly when evaluating individual institutional performance. Second, the retrospective observational design precludes causal inferences regarding differences in outcomes between hospitals. Benchmarking should therefore be regarded as a screening strategy to identify institutions that may benefit from detailed local audits rather than as definitive evidence of differences in quality of care. Finally, administrative databases inevitably lack detailed physiological, microbiological, and therapeutic information that could further improve risk adjustment. As with any model derived from administrative databases, the study is limited by the information available in discharge coding. Detailed clinical, microbiological, physiological, and therapeutic data were not available, and coding inaccuracies or underreporting may have influenced variable ascertainment. Consequently, the model should be interpreted as a risk-adjustment and benchmarking tool based on epidemiological information rather than a causal model intended to evaluate the prognostic impact of specific clinical or microbiological factors.

In conclusion, RADAR-Septic Shock demonstrates that population-based surveillance using standardized administrative data can provide reliable epidemiological information while simultaneously supporting continuous quality assessment through risk-adjusted benchmarking. By integrating surveillance, auditing, and feedback within the regional Sepsis Code strategy, RADAR-Septic Shock offers a scalable framework for monitoring septic shock care, identifying opportunities for improvement initiatives and informed healthcare planning. The combination of population surveillance with complementary clinical registries focused on process indicators may represent an effective strategy for continuously improving the quality of sepsis and septic shock care.

## Figures and Tables

**Table 1:** Characteristics and outcomes of patients with septic shock included in RADAR-Septic Shock according to year.

Year	2018	2019	2020	2021	2022	2023	2024	2018–2024	*p* Value
Number of episodes	4697	4818	4689	5184	5760	6185	6522	37,855	
Males	2817	2860	2860	3112	3434	3701	3913	18,784	0.728
	59.97%	59.36%	60.99%	60.03%	59.62%	59.84%	60.04%	60.04%	
Females	1880	1957	1829	2032	2323	2481	2609	12,502	
	40.03%	40.62%	39.01%	39.20%	40.33%	40.11%	40.02%	39.96%	
Mean age (males)	68.12	68.43	68.81	69.01	68.63	68.86	68.88	68.70	0.32
Mean age (females)	70.14	69.30	69.32	71.01	70.61	69.33	70.42	70.01	0.003
Mean age (SD)	68.9 (17.1)	68.8 (17.2)	69.0 (16.0)	69.8 (15.9)	69.4 (16.7)	69.1 (16.7)	69.5 (16.4)	69.1 (16.5)	0.021
Origin of infection									
Community-acquired	3719	3817	3437	3829	4563	5101	5435	29,901	<0.001
	79.18%	79.22%	73.30%	73.86%	79.22%	82.47%	83.33%	78.99%	
Hospital-acquired	978	1000	1252	1319	1197	1084	1087	7917	
	20.82%	20.76%	26.70%	25.40%	20.78%	17.35%	16.67%	20.91%	
Source of infection									
Urinary	24.99%	25.06%	27.11%	28.48%	27.47%	27.42%	27.95%	27.02%	<0.001
Pulmonary	23.50%	22.86%	19.64%	18.20%	19.60%	23.30%	23.70%	21.63%	<0.001
Abdominal	19.76%	20.32%	18.53%	19.33%	18.47%	18.45%	17.45%	18.83%	0.005
Device-related	9.84%	10.92%	11.81%	10.82%	10.42%	11.08%	10.61%	10.78%	0.082
Skin and soft tissues	4.90%	4.94%	5.05%	3.77%	4.15%	4.95%	4.40%	4.58%	0.008
Endocarditis	1.51%	1.62%	1.64%	1.26%	1.20%	1.44%	1.27%	1.41%	0.295
Central nervous system	0.89%	1.00%	0.51%	0.43%	0.64%	0.71%	0.51%	0.66%	0.002
Documented MDRM	5.53%	5.79%	6.54%	5.40%	6.18%	6.09%	6.73%	6.07%	0.029
Organ Failure added to shock									
Shock plus 1 OF	34.66%	35.46%	31.84%	33.10%	34.34%	36.60%	35.45%	34.60%	<0.001
Shock plus 2 OF	37.88%	35.46%	38.15%	38.25%	38.26%	36.64%	38.75%	37.99%	
Shock plus 3 or more OF	27.46%	26.49%	30.01%	28.65%	27.40%	26.76%	25.80%	27.41%	
Life support measures									
IMV	28.33%	26.97%	32.74%	30.46%	26.99%	25.04%	23.90	27.48%	<0.001
NIMV-PP	7.94%	7.68%	11.04%	14.33%	10.83%	9.58%	9.53%	10.14%	<0.001
RRT	NR	NR	10.70%	10.99%	9.58%	9.29%	9.85%	10.02%	<0.001
ECMO	0.21%	0.33%	0.87%	1.01%	0.60%	0.64%	0.82%	0.65%	<0.001
Mortality									
In-hospital	1767	1680	1999	2064	2195	2076	2134	13,915	<0.001
	37.62%	34.86%	42.63%	39.81%	38.11%	33.56%	32.72%	36.75%	
Community-acquired	1281	1236	1303	1357	1596	1560	1664	9997	<0.001
	34.44%	32.38%	37.91%	35.44%	34.98%	30.58%	30.61%	33.43%	
Hospital-acquired	486	444	696	707	599	516	470	3918	<0.001
	49.69%	44.40%	55.59%	53.60%	50.04%	47.60%	43.23%	49.49%	
30-day	1965	1981	2302	2382	2517	2499	2525	16,171	<0.001
	41.82%	41.12%	49.09%	46.27%	43.69%	40.40%	38.71%	42.71%	

Categorical variables are presented as number (%) and quantitative variables as mean (SD). SD: standard deviation; MDR: multidrug-resistant; OF: organ failure; NR: not reported; IMV: invasive mechanical ventilation; NIV: non-invasive mechanical ventilation (positive pressure); RRT: renal replacement therapy; ECMO: Extracorporeal Membrane Oxygenation.

**Table 2:** Multivariable mortality prediction model used for risk-adjusted benchmarking in RADAR-Septic Shock (2024) (*n* = 37,855).

Characteristic	OR ^1^	95% CI ^1^	*p* Value
Demographics			
Age (years)	1.04	1.04, 1.05	<0.001
Sex			
Male	Ref	—	—
Female	1.24	1.10, 1.40	<0.001
Comorbidities			
Morbidity burden (GMA)	1.00	1.00, 1.01	<0.001
Cirrhosis	1.93	1.45, 2.56	<0.001
Dementia	2.53	1.93, 3.32	<0.001
Active neoplasm	1.28	1.13, 1.45	<0.001
Multidrug-resistant microorganism	0.64	0.50, 0.82	<0.001
Source of infection			
Digestive tract infection	0.87	0.74, 1.01	0.065
Genitourinary infection	0.54	0.47, 0.62	<0.001
Device-related infection	0.57	0.46, 0.70	<0.001
Diagnosis of septic shock			
Present on admission	Ref	—	—
Developed after admission	1.80	1.54, 2.10	<0.001
Organ failure			
Cardiovascular failure	1.71	1.08, 2.78	0.026
Respiratory failure	0.95	0.60, 1.52	0.8
Neurological failure	0.94	0.78, 1.14	0.6
Hematologic failure	1.21	1.01, 1.44	0.040
Hepatic failure	3.03	2.28, 4.03	<0.001
Renal failure	1.04	0.93, 1.17	0.5
Treatments			
Dialysis	2.03	1.67, 2.46	<0.001
ECMO	2.76	1.49, 5.19	0.001
Invasive mechanical ventilation	2.35	1.49, 3.70	<0.001
Non-invasive mechanical ventilation	1.33	1.10, 1.61	0.003
Admission			
Inter-hospital transfer	0.80	0.67, 0.95	0.013

^1^ OR = odds ratio; CI = confidence interval; AMGs: Adjusted Morbidity Groups; ECMO: Extracorporeal Membrane Oxygenation. Odds ratios were obtained from a multivariable logistic regression model for in-hospital mortality.

## Data Availability

The datasets presented in this article are not readily available because research data originate from a restricted-access population database that is not publicly available and belongs to the Catalan Health Service (Department of Health, Generalitat de Cata-lunya). Requests to access the datasets should be directed to jyebenes@gencat.cat.

## Supplementary Materials

The following supporting information can be downloaded at: https://www.mdpi.com/article/10.3390/medsci14060616/s1, Figure S1: Risk-adjusted mortality across the seven territorial Sepsis Code networks (clusters) (RADAR-Septic Shock benchmark cohort, 2024). Boxes represent the observed-to-expected (O/E) mortality ratio for each cluster, identified by anonymized numeric codes. Values below 1 indicate lower-than-expected mortality and values above 1 indicate higher-than-expected mortality. Green indicates significantly lower-than-expected mortality and red significantly higher-than-expected mortality. Figure S2: Risk-adjusted hospital performance according to hospitals complexity (RADAR-Septic Shock benchmark cohort, 2024). (A) Community hospitals (without ICU); (B) General hospitals (with ICU); (C) High-complexity hospitals. Boxes represent the observed-to-expected (O/E) mortality ratio for each hospital, identified by anonymized alphabetical codes. Values below 1 indicate lower-than-expected mortality, whereas values above 1 indicate higher-than-expected mortality. Green boxes indicate significantly lower-than-expected mortality and red boxes significantly higher-than-expected mortality.
