# Supplementary material for: RADAR-Septic Shock: A Population-Based Surveillance and Risk-Adjusted Benchmarking System for Septic Shock in Catalonia
Source: Med Sci (Basel). 2026 Sep 29;14(6):616. doi: 10.3390/medsci14060616 (PMC13706816; doi:10.3390/medsci14060616)

SUPPLEMENTAL MATERIAL

List of the hospitals that have been analyzed and anonymized (Hospital category: 1 community, 2 General, 3 Tertiary).:

H. Universitari de Bellvitge (3), H. de Viladecans (1), H. Comarcal de l'Alt Penedès (1), H. Residència Sant Camil (2), H. St. Joan de Déu de Martorell (1), H. de Sant Boi (1), Consorci Sanitari de l'Anoia (1), Consorci Sanitari Integral (2), H.U. Germans Trias i Pujol (3), H. de Mataró (2), H. de l'Esperit Sant (1), CSMS - Hospital Sant Jaume de Calella (1), H. Clínic de Barcelona (3), H. General de Granollers (2), H. General de Vic (2), H. U. Sagrat Cor (2), Fundació Privada H. de Mollet (1), H.U. de Tarragona Joan XXIII (3), H. de Tortosa Verge de la Cinta (2), H. U Sant Joan de Reus (2), H. de Sant Pau i Santa Tecla (2), Pius H. de Valls (1), H.U. M. Terrassa (3), C.S. Parc Taulí (3), H. Althaia (2), H. de Sant Bernabé (1), H. de Terrassa (2), H.U. de Girona Dr. Josep Trueta (3), CSMS - H. de Blanes (1), H. de Palamós (1), H. de Figueres (1), H. Provincial Santa Caterina (2), H. U. Vall d'Hebron (3), H. U. Arnau de Vilanova (2), H. Santa Maria (1), H. de la Santa Creu i Sant Pau (3), Hospital del Mar-IMAS (3).

Figure S1 (COMP): Risk-adjusted mortality across the seven territorial Sepsis Code networks (clusters)(RADAR-Septic Shock benchmark cohort, 2024). Boxes represent the observed-to-expected (O/E) mortality ratio for each cluster, identified by anonymized numeric codes. Values below 1 indicate lower-than-expected mortality and values above 1 indicate higher-than-expected mortality. Green indicates significantly lower-than-expected mortality and red significantly higher-than-expected mortality.

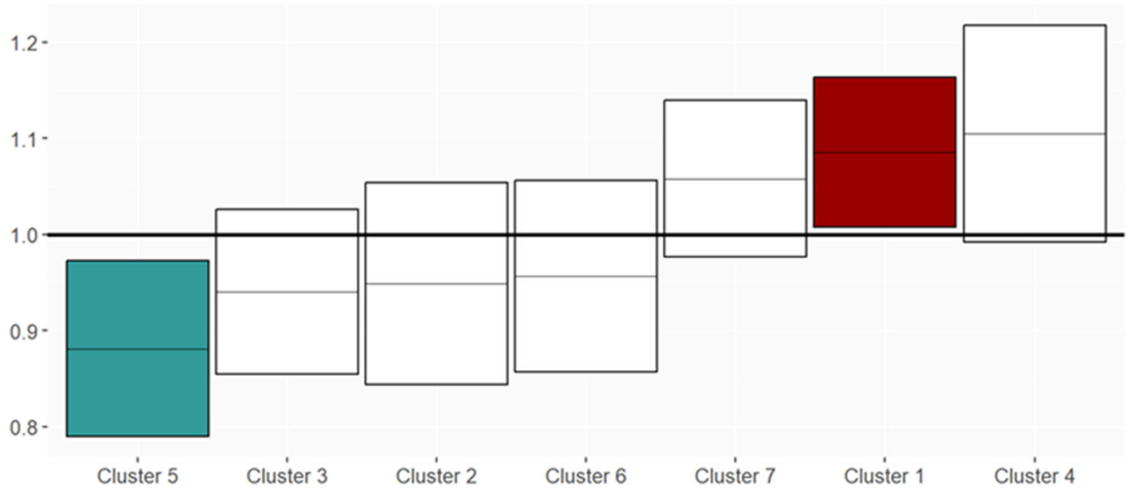

Figure S2 (COMP): Risk-adjusted hospital performance according to hospitals complexity (RADAR-Septic Shock benchmark cohort, 2024). (A) Community hospitals (without ICU); (B) General hospitals (with ICU); (C) High-complexity hospitals. Boxes represent the observed-to-expected (O/E) mortality ratio for each hospital, identified by anonymized alphabetical codes. Values below 1 indicate lower-than-expected mortality, whereas values above 1 indicate higher-than-expected mortality. Green boxes indicate significantly lower-than-expected mortality and red boxes significantly higher-than-expected mortality.

A

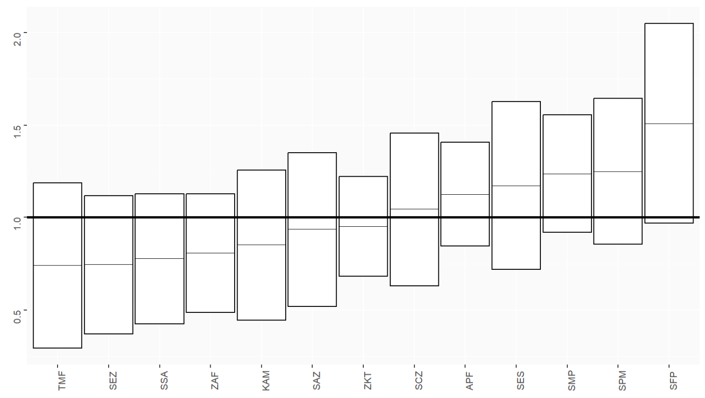

B

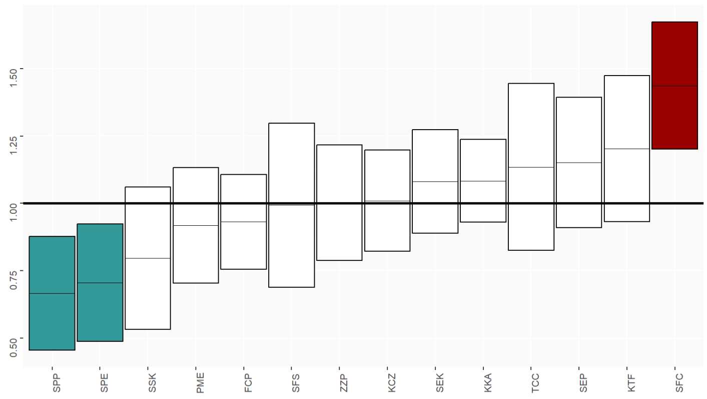

C

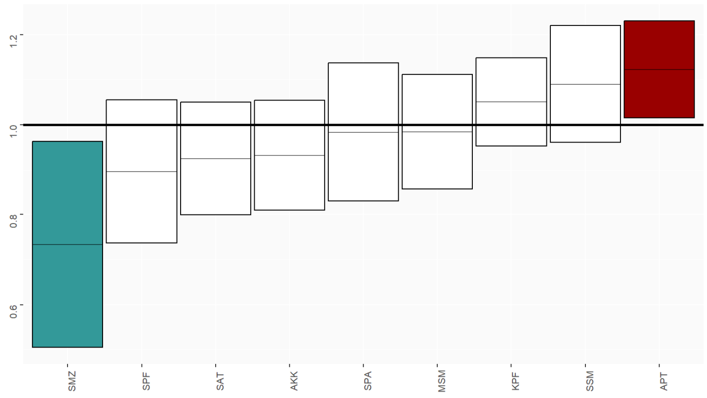

Supplement: Supplementary file 1 [file medsci-14-00616-s001.zip › medsci-4560701-supplementary.pdf]
